# The relationships between photochemical reflectance index (PRI) and photosynthetic status in radish species differing in salinity tolerance

**DOI:** 10.1007/s10265-025-01615-x

**Published:** 2025-01-24

**Authors:** Elsayed Mohamed, Hajime Tomimatsu, Kouki Hikosaka

**Affiliations:** 1 Graduate School of Life Sciences, Tohoku University, Aoba, Sendai, 980-8578 Japan; 2 Botany and Microbiology Department, Faculty of Science, Al-Azhar University, Assuit, 71524 Egypt

**Keywords:** Halophyte, NPQ, Photosynthesis, PRI, Remote sensing, Salinity, Salt tolerance

## Abstract

Since photosynthesis is highly sensitive to salinity stress, remote sensing of photosynthetic status is useful for detecting salinity stress during the selection and breeding of salinity-tolerant plants. To do so, photochemical reflectance index (PRI) is a potential measure to detect conversion of the xanthophyll cycle in photosystem II. *Raphanus sativus* var. *raphanistroides* is a wild radish species closely related to domesticated radish, and is distributed throughout the coastal regions of Japan, where it is thought to be salt tolerant. In this study, we raised wild and domesticated radishes under various salt conditions and assessed growth, photosynthetic status, and PRI. When grown at mild salt stress (50 mM NaCl), wild radish leaves showed photosynthetic activity levels comparable to control plants, whereas the photosynthetic activity of domesticated radish was suppressed. This result suggests that wild radishes are more salt-tolerant than domesticated radishes. Although photosynthetic rate and the photochemical quantum yield were significantly correlated with PRI in both species, the PRI resolution was insufficient to distinguish differences in salt tolerance between wild and domesticated radish. Wild radish had a lower maximum quantum yield (Fv/Fm) when grown under moderate salt stress (200 mM NaCl), suggesting chronic photoinhibition. The relationship between non-photochemical quenching (NPQ) and PRI was significant when leaves with chronic photoinhibition were eliminated but this relationship was not significant when they were included. In contrast, the relationship between photosynthesis and PRI was significant regardless of whether leaves displayed chronic photoinhibition or not. We conclude that PRI is useful to detect relatively large reductions in photosynthetic rate under salinity stress, and that care should be taken to evaluate NPQ from PRI.

**Supplementary Information:**

The online version contains supplementary material available at 10.1007/s10265-025-01615-x.

## Introduction

Soil salinity is a serious abiotic stress that influences the growth and development of plants (Atta et al. 2023; Chaves et al. 2011; Flowers 2004; Mohamed et al. 2015, 2016, 2023). It limits crop yield not only in coastal areas but also in inland areas that suffer from soil sanitization, which are becoming more common due to climate change and/or inappropriate irrigation regimes (Tarolli et al. 2024). The selection or breeding of salt-tolerant crops is therefore necessary to adapt agricultural systems to increasing exposure to salinized areas (van den Burg et al. 2024).

Photosynthesis is one of the most sensitive functions to salinity stress and can therefore be used to assess the salt tolerance of plants (Zahra et al. 2022). Although recent advancements in gas exchange and pulse amplitude modulation methods have facilitated more accurate evaluation of plant photosynthetic status, determining photosynthetic activity still remains time-consuming and can be impossible for large numbers of plants, especially under field conditions. Solar-induced chlorophyll fluorescence and photochemical reflectance index (PRI) are measures that can be tracked using remote sensing techniques, since they offer accurate, quick, and non-destructive methods to assess photosynthetic efficiency under stress conditions (Anderson and Perry 1996; Naumann et al. 2007; Sukhova et al. 2022).

PRI is a normalized reflectance index that uses the ratio of plant reflectance at 531 nm to the reflectance at 570 nm, which acts as a reference (Gamon et al. 1993; Peñuelas et al. 1995). PRI is closely associated with dissipation of excess light energy during photosynthesis. Under non-stressful conditions, most of the light energy absorbed by the chlorophyll molecules in photosystem II (PSII) is used for photochemical electron transfer in the thylakoid membrane. However, under stressful conditions, the absorbed energy cannot be used for photosynthesis due to the suppression of the activity of Calvin-Benson cycle enzymes and/or CO_2_ diffusion to the carboxylation site. Therefore, PSII dissipates excess light energy as heat, since it would otherwise have adverse effects on cellular metabolism and/or structures. The xanthophyll cycle is one of the mechanisms involved in the heat dissipation mechanisms (Goss and Lepetit 2015; Niyogi and Truong 2013). After the onset of stressful conditions, the lumen pH within the thylakoid membrane is reduced and violaxanthin deepoxidase, which converts violaxanthin to antheraxanthin and zeaxanthin, is activated. Zeaxanthin is associated with heat dissipation. Due to the difference in absorbance at 531 nm between violaxanthin and zeaxanthin, the epoxidation status of the xanthophyll cycle is tightly correlated with reflectance at 531 nm (Gammon et al. 1992; Kohzuma and Hikosaka 2018). Many studies have found that PRI is strongly correlated with photosynthetic activity and non-photochemical quenching (NPQ; a measure of heat dissipation) and it may therefore be useful to assess photosynthetic status non-destructively (Filella et al. 1996; Gamon et al. 1992, 1997, 2001; Guo and Trotter 2004; Hikosaka and Tsujimoto 2021; Nichol et al. 2000; Yudina et al. 2020; Zhang et al. 2016).

So far, two studies have tested whether PRI can be useful to detect salinity stress in plants. Naumann et al. (2008) and Zinnert et al. (2012) investigated physiological parameters and PRI in coastal plants subjected to salinity stress. Both studies demonstrated that PRI was strongly correlated with the quantum yield of photosystem II (Ф_P_), thereby suggesting that PRI is a good indicator of salinity stress. However, it remains unclear whether PRI can quantitatively distinguish between plants that are tolerant or sensitive to salinity. If it can, it would be critical for PRI to be useful for screening salt tolerant genotypes.

Salinity stress often causes chronic photoinhibition of PSII (Lu et al. 2023; Wang et al. 2024). Furthermore, PSII is sensitive to and easily inactivated under strong light, but the fast recovery of inactivated PSII can contribute to the maintenance of leaf photosynthetic activity even in stressful environments (Aro et al. 1993). However, if the inactivation rate is too fast to fully recover or the recovery itself is inhibited due to stress, the fraction of active PSII decreases, ultimately leading to a reduced photosynthetic rate (Hikosaka et al. 2004); this is known as photoinhibition (Murata et al. 2007). Using leaves with artificially induced photoinhibition, Hikosaka (2021) showed that light energy partitioning differs between photoinhibited and non-photoinhibited leaves; under mild stress conditions, heat dissipation (NPQ) increases and photosynthesis and chlorophyll fluorescence both decrease as the stress level increases (Flexas et al. 2002; Hikosaka and Noda 2019), but both NPQ and photosynthesis decrease and chlorophyll fluorescence increase with increasing stress in heavily photoinhibited leaves (Hikosaka 2021). Given these results, it is questionable whether the relationship between photosynthetic parameters and PRI holds even when photoinhibited leaves are included in observations.

Radishes, initially cultivated in Europe, are cruciferous vegetables that are now grown as medicinal, forage and oilseed crops throughout the world (Lugasi et al. 2005). The most common varieties of radish include the two domesticated varieties *Raphanus sativus* L. var*. hortensis* and *R. sativus* L. var. *sativus*. Salinity is known to cause severe damage to domesticated radish varieties from Egypt and Italy (Amin 2023; Sanoubar et al. 2020). However, some varieties appear to be tolerant of high salinity. *Raphanus sativus* L. var. *raphanistroides* is a wild radish that is distributed on sandy beaches and other coastal areas throughout Japan (Ishizuka et al. 2020). Since wild radishes can be interbred with domesticated radishes, this species may contribute to the breeding of salt tolerant cultivars of domesticated radishes. Although Sugimoto (2009) demonstrated that Japanese wild radish is not necessarily more tolerant than domesticated radish during germination, to date the growth parameters of wild radishes at other life history stages under salinity conditions have not yet been investigated.

In the present study, we hypothesized that Japanese wild radish is more salt-tolerant than domesticated radish and aimed to address the following questions. First, are there differences in growth and photosynthesis in response to salinity stress between wild and domesticated radish? Second, if so, can we detect these differences using PRI? Third, does the relationship between photosynthetic parameters and PRI hold even when photoinhibited leaves are included in experimental observations?

## Materials and methods

### Materials and growth conditions

To obtain plant material, we collected Japanese wild radish (*R. sativus* var. *raphanistroides*) inflorescences with seeds along the coast near Kamogawa (Chiba Prefecture, Japan, 35°04′55″N, 140°05′46″E) on 6th May, 2019. Plants were found to naturally grow approximately 20 m apart along the coastline. Plant inflorescences were then air dried at room temperature. Seeds were separated by hand and used for subsequent experiments. For domesticated radish (*R. sativus* var. *sativus*), we obtained seeds of the commercial cultivar “Comet” (TAKII and CO., LTD) and used these for subsequent experiments.

Both wild and domesticated radish seeds were sown on pots filled with 1.5 kg of washed river sand. Seedlings were grown for 15 days, then one seedling was transplanted per pot. Pots were subsequently transferred to growth chambers, where they were grown for one month under alternating light conditions (14 h light/10 h dark), at a photosynthetic photon flux density (PPFD) of 200 μmol m^−2^ s^−1^ generated by LED lamps. Growth took place under 25/20 °C temperature conditions to approximate day/night temperatures and at 50% relative humidity. Plants were watered with 50% MS medium prepared using double distilled water. For salinity treatment, 45-day-old radishes were irrigated with 50% MS medium prepared in 50, 100, and 200 mM NaCl for 15 days (Mohamed et al. 2015). We chose these NaCl concentrations because wild radish is considered to be resistant to relatively low NaCl concentrations according to Sugimoto (2009).

### Experiments

We determined gas exchange, spectral reflectance and fluorescence measurements in young, fully developed plant leaves at the same time. For gas exchange parameters, we used a portable photosynthesis system (Li-6400, LiCor, NE, USA) connected with a leaf chamber (2 × 3 cm). An acrylic plate was used as a cover for the topmost portion. The leaf chamber also included two optical fibers to determine fluorescence and the amount of reflected light from the leaves (Tsujimoto and Hikosaka 2021). Chlorophyll fluorescence signals were detected with a fluorometer (PAM2000, Walz, Effetrich, Germany) coupled with one of the optical fibers. Reflection was measured using a spectroradiometer (HR4000, OceanOptics Inc., USA) connected to the other optical fibers (600 µm in diameter) and the device was operated with OceanView spectroscopy software version 1.6.7 (OceanOptics Inc.), with the nonlinear correction being performed by the software.

In the present study, we produced two experimental replicates of plants grown under the same conditions. In the first experiment, time courses of gas exchange, chlorophyll fluorescence parameters and reflectance were assessed once every three days at a CO_2_ concentration of 420 ppm and a temperature of 25 °C during the salt treatment. On each day prior to data collection, plants were incubated in dark conditions for 20–30 min to deactivate heat dissipation (NPQ). Next, the respiration rate, fluorescence in the dark (Fo) and fluorescence during the flash (Fm) were measured. Leaves were then exposed to illumination and the reflectance spectrum was measured immediately, which we treated as the reflectance in the dark. Subsequently, 20–30 min after exposure to illumination (450 μmol m^−2^ s^−1^ PPFD), when the CO_2_ assimilation rate and stomatal conductance (g_s_) values had stabilized, we obtained measurements of gas exchange parameters, fluorescence in the light and in the flash (Fs and F^'^m). For each treatment and each species, four representative plants were used. The maximum quantum yield of photochemistry of PSII in the dark (Fv/Fm), quantum yield in the light (Φ_P_), and NPQ were calculated according to the procedure described by Hikosaka and Tsujimoto (2021).

At the end of the first experiment, four plants were harvested for each treatment and each species, cleaned using tap water and dissected to produce samples of roots, hypocotyls, and leaves. Each of these were dried in an oven at 80 °C and weighed to obtain dry mass (Mohamed et al. 2021).

In the second experiment, we determined the CO_2_ dependence of the gas exchange rate by taking measurements 12–14 days after the onset of salt treatment. The CO_2_ level in the chamber was 420 ppm CO_2_, and then reduced to 300, 200, and 100 for further sequential measurements. Next, the CO_2_ level was returned to 420 ppm, then increased to 600, 800, 1,000, and 1,500 ppm. All measurements were taken with a PPFD of 600 μmol m^−2^ s^−1^ at 25 °C. We did not investigate plants treated with 200 mM NaCl in this experiment due to very low photosynthetic efficiency at this concentration in the first experiment. Once measurements had been obtained, curves were fitted as per the biochemical model of photosynthesis (Farquhar et al. 1980). We first attempted to use the application provided by Sharkey et al. (2007), but failed to obtain reliable values. Therefore, we assumed infinite mesophyll conductance and obtained apparent Vcmax (the maximum rate of rubisco carboxylation) and Jmax (the maximum rate of photosynthetic electron transport) values by fitting to the Ci dependence curve of photosynthesis (A-Ci curve) using a software (KaleidaGraph, Synergy Software, PA, USA).

### Statistical analysis

A two-way analysis of variance (ANOVA) was used to investigate the statistical significance of the association between species, salinity, and their interaction on total biomass, photosynthetic rate, gas and PRI at 15 days after salt treatment for Experiment 1 and on Vcmax and Jmax for Experiment 2. Tukey HSD tests were then used to investigate the statistical significance of pairwise differences in growth and photosynthetic parameter values between different salinity conditions on each day for each species. All statistical analyses were performed using SPSS version 16.0 (IBM SPSS, Armonk, NY, USA).

## Results

### Plant growth

**Fig. 1:**
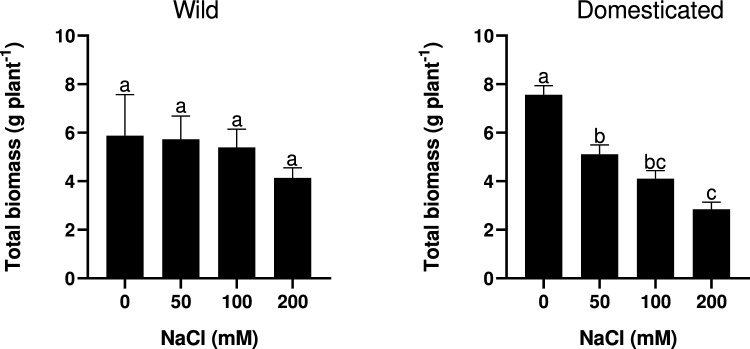
The effect of salinity (0, 50, 100, or 200 mM NaCl) on the total dry mass of wild and domesticated radish after 15 days of salt treatment. Values and bars indicate the mean and standard error of four plants, respectively. Different letters indicate significant differences among treatments in each subspecies as assessed by Tukey HSD test (*P* < 0.05)

Two-way ANOVA results indicated that plant total biomass after salt treatment did not differ between wild and domesticated radish samples (Table 1). Salt treatment appeared to significantly influence total biomass, while the interaction of species and salt treatment was not statistically significant, indicating that the response to salt treatment did not differ between wild and domesticated radish groups. However, the difference in total biomass between treatments was not significant in wild radish whereas it was both greater and statistically significant in domesticated radish (Fig. 1), suggesting that wild radish was relatively more tolerant to salinity stress than domesticated radish.

**Table 1:** Two-way ANOVA results showing *F* and *P* values of the effects of radish subspecies (*i.e.*, wild or domesticated), salinity treatment, and their interaction on total biomass, photosynthetic rate, the maximum carboxylation rate (Vcmax), the maximum electron transport rate (Jmax), stomatal conductance (g_s_), and photochemical reflectance index (PRI)

Parameters	Subspecies	Salinity	Subspecies × salinity
Total biomass	0.467^*ns*^	5.83**	1.59^*ns*^
Photosynthetic rate	3.2*ns*	103.7***	3.46*
Vcmax	70.19***	18.12***	6.56**
Jmax	44.56***	22.76***	5.43*
g_s_	14.4 **	10.6***	1.6^*ns*^
PRI	14.6**	47.7***	33.55***

For biomass, photosynthesis, g_s_ and PRI, data were obtained on Day 15 of the first experiment. For Vcmax and Jmax, data were measured from Days 12–14 of the second experiment

*ns* not significant, *P < 0.05, **P < 0.01, ***P < 0.001

For wild radish, the biomass of each organ (*i.e.*, root, hypocotyl and leaf dry mass) showed non-significant changes at 50 and 100 mM NaCl relative to the control condition, while moderate salinity (200 mM NaCl) led to a significant reduction in both root and hypocotyl biomass (Table 2). Leaf and total biomass dry mass measurements showed non-significant decreases under moderate salinity conditions compared to their respective control conditions. For domesticated radish, both hypocotyl and total biomass significantly decreased under low and moderate salinity levels, while root and leaf biomass showed significant decreases under moderate salinity (200 mM NaCl). Finally, leaf biomass also showed significant reduction under 100 mM NaCl (Table 2).

**Table 2:** The effect of salinity treatment (*i.e.*, 0, 50, 100, or 200 mM NaCl) on the dry weight of root, hypocotyl, and leaf tissue of wild and domesticated radish after 15 days

Salinity	Wild radish			Domesticated radish		
	Root (g plant^−1^)	Hypocotyl (g plant^−1^)	Leaves (g plant^−1^)	Root (g plant^−1^)	Hypocotyl (g plant^−1^)	Leaves (g plant^−1^)
0 mM NaCl	0.65 ± 0.07 a	0.34 ± 0.06 a	6.5 ± 1.69 a	0.305 ± 0.07 a	4.06 ± 0.25 a	3.19 ± 0.33 a
50 Mm NaCl	0.58 ± 0.11 a	0.18 ± 0.02 ab	4.9 ± 0.87 a	0.12 ± 0.03 ab	2.52 ± 0.15 b	2.45 ± 0.26 ab
100 mM NaCl	0.38 ± 0.04 ab	0.21 ± 0.05 ab	4.8 ± 0.66 a	0.13 ± 0.02 ab	2.03 ± 0.07 bc	1.93 ± 0.25 bc
200 mM NaCl	0.19 ± 0.02 b	0.12 ± 0.02 b	3.8 ± 0.41 a	0.07 ± 0.04 b	1.57 ± 0.30 c	1.19 ± 0.07 c

Values indicate mean ± standard error of four plants

Different letters indicate significant differences among treatments in each subspecies as assessed by Tukey HSD test (*P* < 0.05)

### Gas exchange and chlorophyll fluorescence parameters

**Fig. 2:**
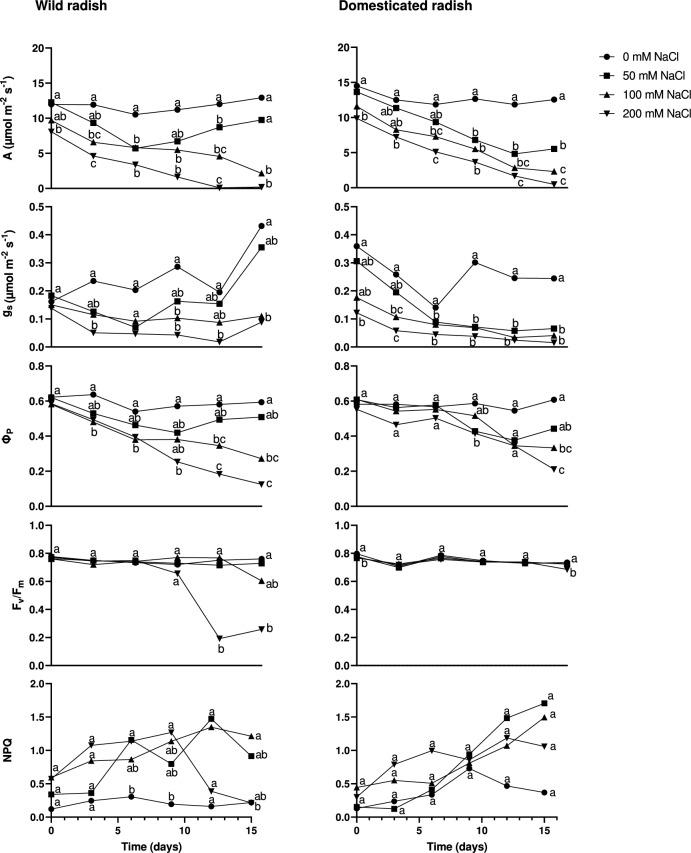
The effect of salinity (0, 50, 100, or 200 mM NaCl) on CO_2_ assimilation rate (A), stomatal conductance (g_s_), the quantum yield of photochemistry in the light (Φ_P_), the maximum quantum yield (Fv/Fm) and non-photochemical quenching (NPQ) for 15 days. Values indicate the means of four plants. Different letters indicate significant differences among treatments in each subspecies as assessed by Tukey HSD test (*P* < 0.05)

In both subspecies, the CO_2_ assimilation rate, g_s_ Φ_P_, Fv/Fm, and NPQ values were found to be relatively stable in control plants (0 mM NaCl) throughout the experiment (Fig. 2). For wild radish plants treated with 100 and 200 mM NaCl, the CO_2_ assimilation rate, g_s_, and Φ_P_ values decreased over time, while those in plants with 50 mM NaCl decreased over time until Day 9, after which they subsequently increased (Fig. 2). Fv/Fm was kept high throughout the treatment in 0 and 50 mM plants, but decreased in the later stages of the 100 and 200 mM treatments (Fig. 2). The NPQ values of salt-treated plants tended to be higher than those of control plants, but the NPQ of 200 mM plants was lower at later stages (Fig. 2). In domesticated radish, the CO_2_ assimilation rate, g_s_, and Φ_P_ values of salt-treated plants tended to be lower than those of control plants and all values decreased over time (Fig. 2). Fv/Fm was at a high level in all plants, and the NPQ levels of salt-treated plants tended to be higher than those of control plants and consistently increased over time (Fig. 2). A two-way ANOVA of values recorded on Day 15 indicated that there were no significant differences in assimilation rate between the two subspecies, but there were significant differences associated with salinity and the interaction of subspecies and salinity (Table 1). One notable difference between subspecies was found in plants treated with 50 mM NaCl. CO_2_ assimilation rate in 50 mM wild radish was relatively high and did not significantly differ from control plants, whereas CO_2_ assimilation rates in 50 mM domesticated radish were much lower than those in control plants. Two way-ANOVA results at Day 15 indicated that g_s_ was significantly influenced by subspecies and salt treatment, but not by their interaction (Table 1).

**Fig. 3:**
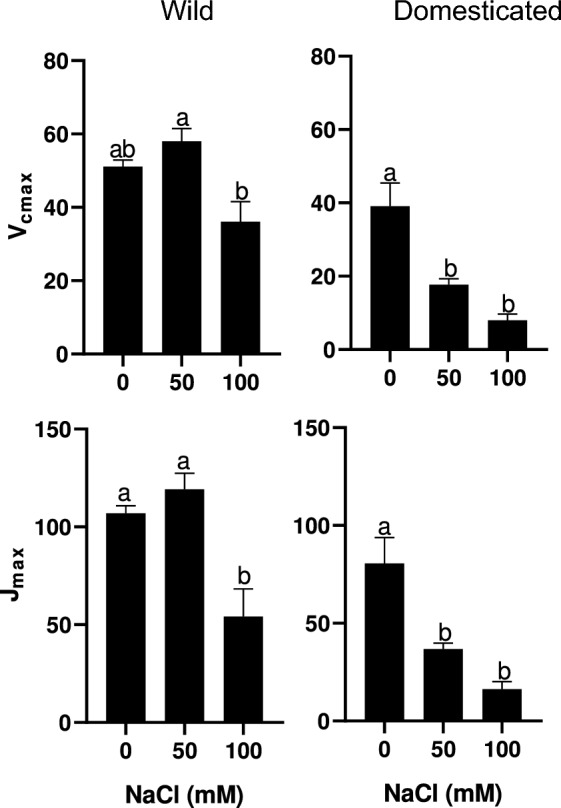
The maximum rate of carboxylation (V_cmax_) and maximum rate of electron transport (J_max_) in wild and domesticated radish plants subjected to 0, 50, and 100 mM NaCl after 12 days of treatment. Values and bars indicate the mean and standard error of four plants, respectively. Different letters indicate significant differences among treatments in each subspecies as assessed by Tukey HSD test (*P* < 0.05)

Further analysis of two-way ANOVA results showed that both Vcmax and Jmax were significantly influenced by subspecies, salinity, and the interaction of subspecies and salinity (Table 1). For example, the Vcmax and Jmax values of 50 mM wild radish were comparable to those of control plants, whereas those of 50 mM domesticated radish plants were much lower (Fig. 3). Finally, the Vcmax and Jmax values of 100 mM plants were all much lower than those of control plants in both subspecies.

### PRI and photosynthetic activity

**Fig. 4:**
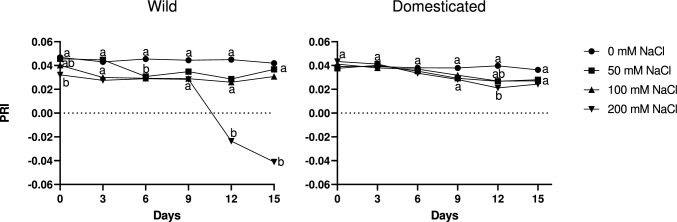
The effect of salinity treatment (0, 50, 100, and 200 mM NaCl) on photochemical reflectance index (PRI) for 15 days. Values indicate the mean of four plants. Different letters indicate significant differences among treatments in each subspecies as assessed by Tukey HSD test (*P* < 0.05)

PRI values ranged between −0.05 and 0.05 depending on salt treatments and on subspecies. In the control groups of both subspecies, PRI values tended to be stable and were not lower than salt-treated plants throughout the treatment (Fig. 4). In wild radish, PRI values were lower in 100 and 200 mM plants and 200 mM plants exhibited very low values at later stages. In contrast, in domesticated radish, PRI values in all salt-treated plants tended to decrease with time. A two-way ANOVA for Day 15 revealed that PRI values were significantly influenced by subspecies, salinity treatment, and their interaction (Table 1). However, these trends were not necessarily statistically significant when assessed using Tukey HSD tests (Fig. 4). For example, in wild radish, photosynthetic rates differed between control and 100 mM NaCl plants in most cases during the salinity treatment, yet PRI differed only on Day 6 (Fig. 4). Similarly, in domesticated radish, PRI did not significantly differ between control and 100 mM NaCl plants during the treatment (Fig. 4).

**Fig. 5:**
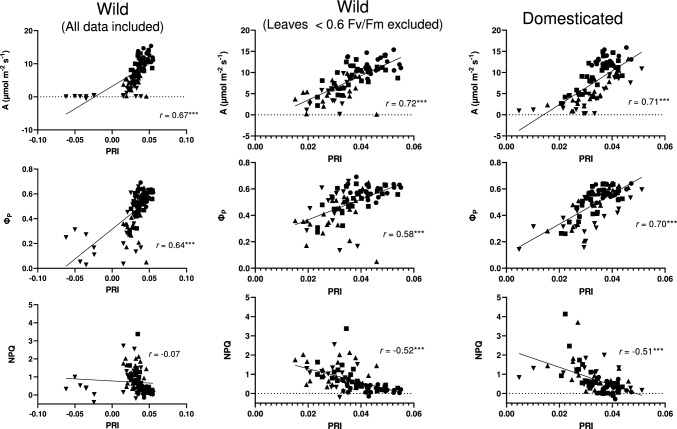
Relationship between photochemical reflectance index (PRI) and CO_2_ assimilation rate (A), the quantum yield of photochemistry in the light (Φ_P_), and non-photochemical quenching (NPQ) in wild and domesticated radish plants grown under different salinity treatments (0, 50, 100, and 200 mM NaCl). All data from the first experiment (time course) were pooled except for the middle column, in which data from the leaves with Fv/Fm < 0.6 were eliminated

Finally, we examined correlations between photosynthetic parameters and PRI (Fig. 5). We found that PRI was significantly correlated with CO_2_ assimilation rate and Φ_P_ in both subspecies (Fig. 5). PRI was correlated with NPQ in domesticated radish, but not in wild radish (Fig. 5). Since it is known that leaves under chronic photoinhibition show a different energy allocation in PSII from non-stressed leaves (Hikosaka 2021), we eliminated leaves that had Fv/Fm values < 0.6 in wild radish plants treated with 100 or 200 mM NaCl, then reexamined these correlations. When the leaves with lower Fv/Fm were excluded, the correlation between NPQ and PRI was significant in wild radish (Fig. 5).

## Discussion

Overall, our results demonstrated that wild radish is more tolerant to salinity than domesticated radish. At 50 mM NaCl, the photosynthetic rate in wild radish was comparable to that of control plants, whereas the photosynthetic rate in domesticated radish was much lower. Stomatal conductance, Vcmax, and Jmax values were also similar between the 0 and 50 mM NaCl treatments in wild radish, indicating that both biochemical activity and CO_2_ diffusion were not influenced by the mild 50 mM NaCl treatment in wild radish. This result contrasted with those of domesticated radish, in which all stomatal conductance, Vcmax, and Jmax values were suppressed at 50 mM. Notably, this pattern has generally been observed in glycophyte species (Naumann et al. 2007; Rasouli et al. 2021). A time course comparison of the photosynthetic rate demonstrated that photosynthesis was suppressed by 50 mM NaCl in wild radish during the early stages of salinity treatment, but plants recovered by the later stage, suggesting that acclimation to salinity stress occurs in wild radish. Although the two-way ANOVA suggested that salinity response of total biomass was not different between wild and domesticated radish (*i.e.*, the interaction of treatment and subspecies was not significant), this result may be due to the relatively short period of the experiment; we would observe larger differences in salinity response between the subspecies, if the experiment were conducted for a longer period. Therefore, we can conclude that wild radish is more tolerant to low salinity treatment than domesticated radish. However, both photosynthesis and growth were suppressed at 100 and 200 mM NaCl in both subspecies, suggesting that wild radish is not tolerant to moderate or severer salinity stress.

When leaves with lower Fv/Fm were eliminated, the PRI values of both subspecies were found to be significantly correlated with photosynthetic parameters (*i.e.*, photosynthetic rate, Φ_P_, and NPQ) across all salt treatments, a finding that agrees with those of previous studies (Naumann et al. 2008; Zinnert et al. 2012). These results suggest that PRI may be useful to assess salinity tolerance in plants. Since PRI can be obtained from many individual plants if a hyperspectral camera is used (Kohzuma et al. 2021; Ogawa et al. 2024), it may also be useful for screening salt tolerant plants during selection and breeding. However, the determination coefficient (r^2^) was around 0.5, which is not high. Furthermore, Tukey HSD tests for PRI were not able to distinguish differences between treatments when they were observed in photosynthetic rate or Φ_P_ due to the large experimental variance in PRI values. These results suggest that the resolution required to assess salt tolerance for PRI is lower than that for other measurements such as gas exchange or chlorophyll fluorescence. PRI may therefore be used to assess salt tolerance only when the degree of photosynthetic suppression is large.

During the later stages of salt treatment, we found that Fv/Fm was suppressed in 200 mM wild radish, meaning that the inactivation of PSII had not fully recovered following 20–30 min in the dark in these plants. This suggests chronic photoinhibition in PSII (Müller et al. 2001). There are two contrasting interpretations of this phenomenon. First, wild radish may be very susceptible to 200 mM NaCl. That is, due to suppression in energy utilization in Calvin-Benson cycle or electron transport induced by salt stress, excess energy results in the production of reactive oxygen species, thereby leading to enhanced PSII photoinactivation and the suppressed recovery of photodamaged PSII (Kato et al. 2002; Murata et al. 2007; Oguchi et al. 2009). Second, wild radish may use inactivated PSII as a quencher of excess energy to protect active PSIIs (Critchley and Russell 1994; Kato et al. 2002; Krause 1988; Lee et al. 2001; Öquist et al. 1992). In 200 mM wild radish, fluorescence yield in the dark (Fo) was slightly lower than in other plants when Fv/Fm values were low (Day 12 and 15; Fig. S1). This result is consistent with the latter hypothesis; fluorescence yield might be reduced because of the increased heat dissipation in the photoinactivated PSII. If this hypothesis is true, the accumulation of inactivated PSII might partly alleviate further damage to PSII.

Under chronic photoinhibition, NPQ can be divided into two (or more) components (Müller et al. 2001; Quick and Stitt 1989). One is fast relaxation NPQ (NPQ_F_), which is mainly explained by energy-dependent quenching (qE), while the other is slow relaxation NPQ (NPQ_S_), which is mainly explained by chronic photoinhibition (qI). Hikosaka (2021) investigated energy partitioning and PRI in leaves with artificially-induced photoinhibition, and found that NPQ_S_ exhibited a parabolic curve against the degree of photoinhibition (as represented by Fv/Fm), and was greatest under intermediate photoinhibition. In contrast, NPQ_F_ was lower in leaves with a greater degree of photoinhibition. In the present study, we did not estimate NPQ_S_ due to technical limitations and thus the estimated NPQ included only NPQ_F_. This may partly explain the decrease in NPQ observed in the late stages in wild radish treated with 200 mM salt treatment; it might dissipate excess light energy via NPQ_S_ rather than NPQ_F_. Hikosaka (2021) also observed that PRI was correlated with total NPQ but not with NPQ_F_. This also explains why NPQ was not correlated with PRI when photoinhibited leaves (*i.e.*, those with low Fv/Fm values) were included in the wild radish dataset. In this case we may need to be careful when the NPQ of photoinhibited leaves is assessed using PRI.

However, we also found that PRI was significantly correlated with photosynthetic rate and Φ_P_ irrespective of chronic photoinhibition of measured leaves. Lower PRI values in photoinhibited leaves may be due to more abundant zeaxanthin in photoinhibited leaves. In leaves without chronic photoinhibition, excess energy is dissipated only by NPQ_F_, whereas in leaves with chronic photoinhibition, excess energy can be dissipated by NPQ_F_ or NPQ_S_. Compared with leaves without chronic photoinhibition, leaves subjected to chronic photoinhibition might dissipate more energy, and consequently there would be greater suppression of the energy allocated to photosynthesis. Furthermore, since PRI is correlated with total NPQ rather than NPQ_F_ (Hikosaka 2021), PRI is associated with photosynthetic activity even when photoinhibited leaves are included in the sample examined.

Finally, since wild radish can be interbred with domesticated radish, we speculate that it may be possible to produce salinity-tolerant radish varieties via crossing. Moreover, the breeding of salinity-tolerant cultivars may contribute to improvement in the agricultural productivity of salinized land. However, we also note that wild radish was not necessarily tolerant to moderate salinity (100–200 mM NaCl), indicating that its salinity tolerance may be weaker than other halophyte species such as *Chenopodium quinoa* and *Beta maritima* (Rasouli et al. 2021). This suggests that the introgression of wild radish traits may result in tolerance only to mild salinity stress (< 100 mM NaCl).

## Conclusion

Our results indicate that wild radish is more tolerant to mild salinity stress than domesticated radish, especially following acclimation to salinity conditions, since it maintained a high level of biochemical activity and CO_2_ diffusion. PRI was significantly correlated with both photosynthetic rate and the quantum yield of photochemistry across salinity conditions in both subspecies, indicating that it may be a useful measure for assessing salinity tolerance using remote sensing. However, the resolution of PRI values was low due to the relatively large variance present in the dataset, meaning that PRI can be used only when the photosynthetic rate is very different. Our results demonstrated that PRI is related with NPQ when leaves with chronic photoinhibition are eliminated. However, when these leaves are included, we found that PRI is not correlated with NPQ. This may be because PRI is related to total NPQ rather than the fast-relaxation component of NPQ. However, correlations between photosynthetic activity (*i.e.*, photosynthetic rate and the quantum yield of photochemistry) and PRI were significant regardless of the inclusion of photoinhibited leaves, suggesting that PRI is useful for assessing photosynthetic status in plants subjected to salinity stress.

## Supplementary Information

Below is the link to the electronic supplementary material.

Supplementary file1 (PDF 81 KB)
