# Supplementary material for: The relationships between photochemical reflectance index (PRI) and photosynthetic status in radish species differing in salinity tolerance
Source: J Plant Res. 2025 Jan 24;138(2):231–41. doi: 10.1007/s10265-025-01615-x (PMC11910433; doi:10.1007/s10265-025-01615-x)
Supplement: Supplementary file 1 — Supplementary file1 (PDF 81 KB) [file 10265_2025_1615_MOESM1_ESM.pdf]

Electronic supplementary material

Title: The relationships between photochemical reflectance index (PRI) and photosynthetic status in radish species differing in salinity tolerance

Authors: Elsayed Mohamed, Hajime Tomimatsu, Kouki Hikosaka

Journal: Journal of Plant Research

Corresponding author

Kouki Hikosaka, Graduate School of Life Sciences,

Tohoku University, Aoba, Sendai 980-8578, Japan.

Email: hikosaka@tohoku.ac.jp

Content:

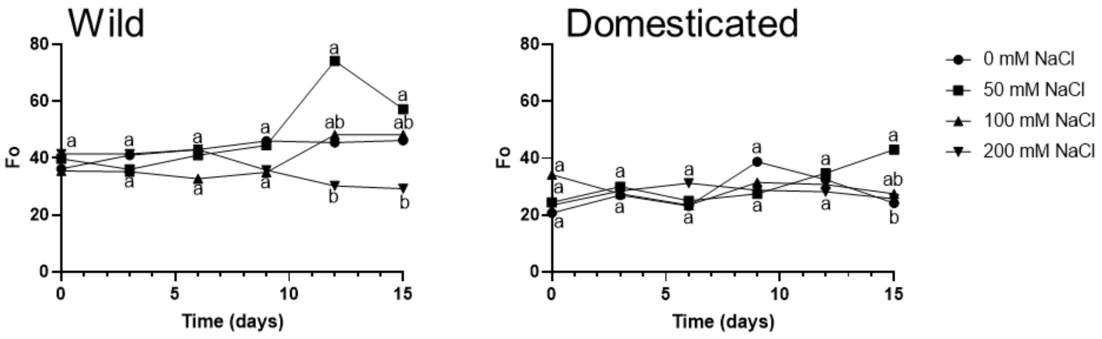

Fig. S1 The effect of salinity (0, 50, 100, or 200 mM NaCl) on the fluorescence signal in the dark (Fo) for 15 days. Values indicate the means of four plants. Different letters indicate significant differences among treatments in each subspecies as assessed by Tukey HSD test ($P < 0.05$)
